# Bioprocessing optimization for efficient simultaneous removal of methylene blue and nickel by *Gracilaria* seaweed biomass

**DOI:** 10.1038/s41598-020-74389-y

**Published:** 2020-10-15

**Authors:** Noura El-Ahmady El-Naggar, Nashwa H. Rabei

**Affiliations:** Department of Bioprocess Development, Genetic Engineering and Biotechnology Research Institute, City of Scientific Research and Technological Applications (SRTA-City), Alexandria, 21934 Egypt

**Keywords:** Metals, Environmental biotechnology, Applied microbiology, Biotechnology, Environmental sciences

## Abstract

The pollution of water by heavy metal ions and dyes, particularly from industrial effluents, has become a global environmental issue. Therefore, the treatment of wastewater generated from different industrial wastes is essential to restore environmental quality. The efficiency of *Gracilaria* seaweed biomass as a sustainable biosorbent for simultaneous bioremoval of Ni^2+^ and methylene blue from aqueous solution was studied. Optimization of the biosorption process parameters was performed using face-centered central composite design (FCCCD). The highest bioremoval percentages of Ni^2+^ and methylene blue were 97.53% and 94.86%; respectively, obtained under optimum experimental conditions: 6 g/L *Gracilaria* biomass, initial pH 8, 20 mg/L of methylene blue, 150 mg/L of Ni^2+^ and 180 min of contact time. Fourier Transform Infrared Spectroscopy (FTIR) spectra demonstrated the presence of methyl, alkynes, amide, phenolic, carbonyl, nitrile and phosphate groups which are important binding sites involved in Ni^2+^ and methylene blue biosorption process. SEM analysis reveals the appearance of shiny large particles and layers on the biosorbent surface after biosorption that are absent before the biosorption process. In conclusion, it is demonstrated that the *Gracilaria* seaweed biomass is a promising, biodegradable, ecofriendly, cost-effective and efficient biosorbent for simultaneous bioremoval of Ni^2+^ and methylene blue from wastewater effluents.

## Introduction

Today, heavy metals, pigments and dyes represent common and dangerous pollutants in wastewaters generated from different industries like the pulp and paper, dyeing, as well as textile industries^1^. The heavy metals are one of the most toxic environmental contaminants due to rapid industrial activities development and increased population in the world. Contamination with different heavy metals is a global environmental concern. Metal toxicity is of great concern due to their non-biodegradable properties, bioaccumulation and toxicological impacts on the human health and ecosystems even in low concentrations^2^. Different industries such as iron and steel, metallurgy, electrolysis, fertilizer and pesticide industry, energy and fuel production, electroplating, photography, leather working, mining, atomic energy installation and aerospace etc. generate and discharge wastes containing various heavy metals into the fresh water resources. Yaseen and Scholz^3^ reported that the main heavy metals of real textile wastewater, that causes environmental concerns, are zinc, lead, cadmium and nickel.

Nickel (II) is present in the wastewater of the fertilizers, mining, metallurgical, paper, pigments, battery, nickel electroplating, galvanizing, stainless steel, ceramic and aircraft industries. Nickel (II) is an essential micronutrient and/or cofactor and is recognized as a component in of many enzymes involved in significant metabolic processes like the metabolism of hydrogen, ureolysis, acidogenesis, methane biogenesis^4^, essential for the regulation of hormones and protein metabolism in the human body^5^. The permissible nickel concentration should not exceed 0.02 mg/L “as suggested by the World Health Organization”^6^.

Human exposure to elevated concentrations of nickel over the permissible levels leads to nickel accumulation in the lymph nodes that increase the risk of cancer^7^ and is associated with different types of chronic and acute disorders such as vomiting, nausea, diarrhea, kidney edema, pulmonary fibrosis, shortness of breath, dermatitis in the skin, chest pain, nephrotoxicity, hepatotoxicity, neurotoxicity, gene toxicity and inhibition of oxidative enzymes activity^4,5^.

Dyes are another group of hazardous substances, enormous amounts of commercial dyes are commonly used in several industries like cosmetic, leather, printing, food, plastic, rubber, pharmaceuticals and textile industries^8^. As a result, these industries generate huge quantities of undesirable colored wastewater which is highly contaminated with dyes^9^ and discharged in effluents^10^. The wastewaters discharged from textile factories contain amounts of the dyes (very commonly methylene blue and methyl orange) mixed with heavy metals (nickel mainly from the organo-metallic dyes, copper and cadmium) and other pollutants^1^. The discharges of dyes into effluents cause harmful effects on the organisms in the aquatic ecosystem and food chain^11^. Dyes have non-biodegradable characteristics, are generally resistant in nature, stable to heat, light and the oxidizing agents^12^. A combination of dyes, metals and other contaminants can be found in the wastewater discharged from textile factories.

**Figure 1:**
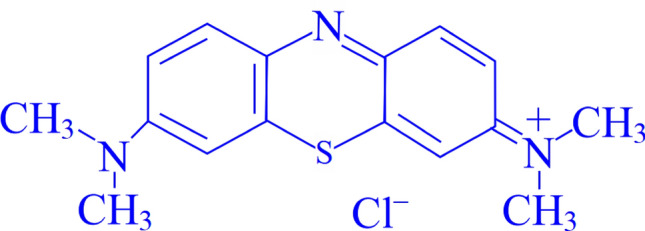
Formula of methylene blue.

Methylene blue is an important heterocyclic aromatic basic dye with the chemical formula C_16_H_18_N_3_SCl. Its structure is shown in Fig. 1. The methylene blue is mainly used as a textile dye to color wool, cotton, silk and tannin printing. The methylene blue is also used for the treatment of fungal infections in aquaculture. The methylene dye has a variety of adverse effects on human beings, despite several beneficial uses^13^. The severe side effects (if the dye is swallowed) include vomiting, nausea, gastrointestinal tract pain, and diarrhea. If inhaled, it can cause chest pain, dizziness, trouble breathing, tachycardia, high fever, mental confusion, headache, convulsions, pale or blue skin, mild bladder irritation, increased sweating, methemoglobinemia and anemia. Methylene blue dye can also cause temporary or permanent burns in the eyes of humans and other animals and skin irritation^14,15^.

Due to their adverse effects, removal of methylene blue dye and heavy metals from wastewaters are essential. Metals and dyes pollutants can be removed from aqueous solutions and wastewater effluents by diverse conventional physico-chemical methods of treatments such as coagulation, flocculation, electrochemical methodology, oxidation, filtration, solvent extraction, reverse osmosis, membrane separation, ion-exchange, precipitation and biological treatments^16^. However, all these approaches have drawbacks and limitations to their use, as they are not always efficient, expensive and also generation of large amounts of toxic solid wastes (not eco-friendly)^17^.

Biosorption is an effective method for treatment of wastewater^18^ by removing toxic metal ions and dyes pollutants from hazardous effluents at low costs using natural biosorbents. Marine algae (seaweeds) is renewable natural biomass, have been shown to be extremely efficient biosorbents with a high binding capacity to a variety of metals and dyes pollutants in aqueous effluents^19^. Dried biomass of marine algae are promising as an biosorbents for metal removal^20,21^ or dyes removal from wastewater. Biosorption by biomass of algae has mainly been occurring through interactions with cell wall^22^. This is attributed to the existence of various cell wall functional groups like sulfate, carboxyl hydroxyl, and amino groups that can act as cell surface binding sites for pollutants. The main mechanisms of binding, including adsorption, ion exchange, complex formation between ligands on the surface of the seaweeds and pollutants cations, chelation, surface precipitation or diffusion interior the cells, bioaccumulation inside cells and binding to proteins and other intracellular components^23,24^.

The goals of this study were to assess the potential of the dried biomass of marine red algae *Gracilaria* as biosorbent for simultaneous bioremoval of methylene blue and nickel from aqueous solutions, to achieve the optimum conditions for biosorption process using FCCCD and to characterize the biosorbent.

## Results and discussion

Several studies have been performed on the adsorption of only one contaminant, despite the fact that both dyes and metals are present together in large quantities of industrial wastewaters^25,26^. Therefore, high priority was given to the simultaneous removal of heavy metal ions and dyes in wastewater treatment processes^27^.

In the current study, the dried biomass of marine red algae *Gracilaria* was used as biosorbent for simultaneous bioremoval of methylene blue and nickel from aqueous solutions. *Gracilaria* is a genus of red algae (Rhodophyta). In the current study, *Gracilaria verrucosa* had been used because it is a cost effective biosorbent, very abundant macroalgae in marine areas and easily collected. Very scarce studies have been done on the biosorption by *G. verrucosa*^23^. The red algae have a complex cell walls consist of mannan fibrils, xylan, cellulose, as well as a large polysaccharide matrix which contain gelans, agar, carrageen and protein, which are of economic importance along with minerals. Calcium can be absorbed by some red seawater algae and stored in their bodies as calcium carbonate^28,29^. These cell wall components and calcium carbonate can provide various functional groups, increasing the metal ions and dye binding sites on the algal surface.

### Statistical optimization of simultaneous bioremoval of methylene blue and nickel by *Gracilaria* seaweed biomass using FCCCD

Different variables can affect the biosorption processes such as biosorbent dosage, contact time, temperature, pH of the solution and initial concentration of the metal ions^30,31^. Optimizing the conditions for the biosorption process is crucial to achieving maximum biosorption. The conventional single variable optimization method has been used for optimization of the process, but this method is not only laborious, expensive and time consuming, but also it does not reflect the impact of interactions between the independent process factors^32^. The optimization of process parameters by statistical experimental designs has been used for many decades. Mathematical and statistical techniques such as response surface methodology (RSM) are very advantageous as they help to determine the optimum level of each variable and help to explore the interactions between different variables for maximum response^33^.

Optimization of biosorption process parameters was carried out using FCCCD to study the individual, quadratic and interaction impacts among the process parameters including algal biomass concentration, initial pH level, nickel concentration, contact time and methylene blue concentration on simultaneous bioremoval of Ni^2+^ ions and methylene blue from aqueous solution by dry *Gracilaria* seaweed biomass. FCCCD was also used to predict the best working conditions for the biosorption process for simultaneous bioremoval of methylene blue and Ni^2+^. The FCCCD had eight center points, 32 factorial and 10 axial points which resulted in a total of 50 experimental trials that were used for optimization of the selected variables. In order to determine experimental errors, the eight replicates at the center points were performed. Table 1 shows a design matrix containing coded and actual values of the five process parameters, the experimental and predicted removal percentages of methylene blue and Ni^2+^, and the residuals. Based on the variations in the levels of the five process parameters, the experimental results show substantial differences in the removal percentages of both methylene blue and Ni^2+^. Removal percentage of methylene blue ranged from 52.78 to 94.86% and the removal percentage of Ni^2+^ ions ranged from 58.33 to 97.53%.

**Table 1:** Face-centered central composite design matrix of simultaneous biosorption of methylene blue and nickel by using *Gracilaria* seaweed biomass.

Std	Run	Point Type	X_1_	X_2_	X_3_	X_4_	X_5_	Methylene blue removal (%)			Nickel removal (%)		
								Actual	Predicted	Residuals	Actual	Predicted	Residuals
31	1	Fact	− 1	1	1	1	1	82.93	83.30	− 0.38	81.59	80.67	0.92
40	2	Axial	0	0	0	1	0	91.66	92.13	− 0.47	92.58	92.65	− 0.07
12	3	Fact	1	1	− 1	1	− 1	83.09	82.70	0.39	80.57	79.62	0.95
22	4	Fact	1	− 1	1	− 1	1	73.27	73.90	− 0.62	80.19	79.64	0.56
44	5	Center	0	0	0	0	0	91.12	92.09	− 0.97	94.50	94.77	− 0.27
9	6	Fact	− 1	− 1	− 1	1	− 1	65.88	65.70	0.19	68.24	69.08	− 0.84
48	7	Center	0	0	0	0	0	91.94	92.09	− 0.15	93.08	94.77	− 1.69
15	8	Fact	− 1	1	1	1	− 1	81.24	81.02	0.21	79.06	79.89	− 0.82
18	9	Fact	1	− 1	− 1	− 1	1	73.80	73.19	0.61	74.39	74.22	0.17
16	10	Fact	1	1	1	1	− 1	85.80	86.35	− 0.55	80.13	80.56	− 0.43
38	11	Axial	0	0	1	0	0	89.76	89.88	− 0.12	93.96	94.25	− 0.28
43	12	Center	0	0	0	0	0	92.70	92.09	0.61	95.38	94.77	0.61
32	13	Fact	1	1	1	1	1	81.06	81.01	0.05	80.49	80.47	0.02
30	14	Fact	1	− 1	1	1	1	85.64	85.74	− 0.09	91.58	90.95	0.63
4	15	Fact	1	1	− 1	− 1	− 1	77.92	78.05	− 0.13	79.08	80.07	− 0.99
39	16	Axial	0	0	0	− 1	0	83.86	84.55	− 0.69	87.99	87.69	0.29
35	17	Axial	0	− 1	0	0	0	86.54	87.36	− 0.82	89.80	88.93	0.87
20	18	Fact	1	1	− 1	− 1	1	81.09	81.70	− 0.61	85.78	84.13	1.64
19	19	Fact	− 1	1	− 1	− 1	1	80.44	81.13	− 0.69	83.15	83.51	− 0.36
41	20	Axial	0	0	0	0	− 1	90.72	90.77	− 0.05	93.40	92.17	1.23
27	21	Fact	− 1	1	− 1	1	1	82.76	82.87	− 0.11	80.34	81.09	− 0.75
8	22	Fact	1	1	1	− 1	− 1	81.26	81.97	− 0.71	82.47	81.52	0.95
5	23	Fact	− 1	− − 1	1	− 1	− 1	64.89	65.66	− 0.77	67.07	67.52	− 0.45
47	24	Center	0	0	0	0	0	92.91	92.09	0.81	95.97	94.77	1.20
45	25	Center	0	0	0	0	0	92.36	92.09	0.27	91.50	94.77	− 3.27
49	26	Center	0	0	0	0	0	93.73	92.09	1.64	96.34	94.77	1.57
26	27	Fact	1	− 1	− 1	1	1	84.78	85.30	− 0.52	85.36	86.04	− 0.68
23	28	Fact	− 1	1	1	− 1	1	82.26	81.84	0.42	84.48	83.60	0.88
46	29	Center	0	0	0	0	0	93.08	92.09	0.99	95.72	94.77	0.95
10	30	Fact	1	− 1	− 1	1	− 1	78.69	78.77	− 0.08	74.86	75.12	− 0.26
42	31	Axial	0	0	0	0	1	94.86	95.97	− 1.11	97.53	98.53	− 1.00
2	32	Fact	1	− 1	− 1	− 1	− 1	64.98	65.08	− 0.10	61.69	62.28	− 0.58
25	33	Fact	− 1	− 1	− 1	1	1	79.94	79.84	0.10	81.76	80.87	0.89
21	34	Fact	− 1	− 1	1	− 1	1	73.70	73.97	− 0.27	76.51	77.19	− 0.69
17	35	Fact	− 1	− 1	− 1	− 1	1	70.08	69.07	1.01	69.53	69.99	− 0.46
36	36	Axial	0	1	0	0	0	92.91	93.26	− 0.35	93.74	94.38	− 0.65
34	37	Axial	1	0	0	0	0	87.77	88.40	− 0.62	88.66	89.29	− 0.63
3	38	Fact	− 1	1	− 1	− 1	− 1	70.93	69.87	1.06	77.68	78.57	− 0.90
33	39	Axial	− 1	0	0	0	0	83.13	83.67	− 0.54	87.69	86.85	0.85
50	40	Center	0	0	0	0	0	93.56	92.09	1.46	94.78	94.77	0.01
24	41	Fact	1	1	1	− 1	1	78.98	78.21	0.77	81.13	82.45	− 1.32
13	42	Fact	− 1	− 1	1	1	− 1	77.97	77.74	0.24	78.80	78.90	− 0.10
28	43	Fact	1	1	− 1	1	1	85.77	84.78	1.00	82.53	82.67	− 0.14
14	44	Fact	1	− 1	1	1	− 1	86.68	86.61	0.07	83.47	83.16	0.31
6	45	Fact	1	− 1	1	− 1	− 1	74.36	73.19	1.17	70.63	70.83	− 0.20
29	46	Fact	− 1	− 1	1	1	1	84.93	84.47	0.45	87.27	87.56	− 0.29
1	47	Fact	− 1	− 1	− 1	− 1	− 1	52.78	53.35	− 0.56	58.33	57.19	1.14
11	48	Fact	− 1	1	− 1	1	− 1	72.69	73.18	− 0.49	77.84	77.17	0.67
37	49	Axial	0	0	− 1	0	0	84.56	85.61	− 1.05	90.68	90.18	0.50
7	50	Fact	− 1	1	1	− 1	− 1	78.11	77.98	0.13	82.13	81.80	0.33

Variable	Variable code	− 1	0	1									
Algal biomass (g/L)	X_1_	2	6	10									
Initial pH level	X_2_	6	8	10									
Nickel conc. (mg/L)	X_3_	100	150	200									
Methylene blue conc.(mg/L)	X_4_	10	20	30									
Contact time (min)	X_5_	60	120	180									

The highest removal percentages of Ni^2+^ and the methylene blue were achieved in the run no. 31 with percent of 97.53% and 94.86% for Ni^2+^ ions and the methylene blue; respectively, where algal biomass concentration 6 g/L, initial pH was 8, Ni^2+^ concentration was 150 mg/L, the methylene blue concentration was 20 mg/L and contact time was 180 min at room temperature. On the other hand, the minimum removal percentages of Ni^2+^ ions and methylene blue were observed in run number 47 where algal biomass concentration 2 g/L, initial pH was 6, Ni^2+^ concentration was 100 mg/L, the methylene blue concentration was 10 mg/L for 60 min of contact time at 30 °C. The adsorption efficiency decreased in the run number 47 may be as a result of low level of algal biomass or contact time.

### Multiple regression analysis and ANOVA

Both nickel removal (%) and methylene blue removal (%) data were statistically analyzed by using multiple regression analysis and the results were presented in Tables 2, 3, 4 and 5. Linear, interactions and quadratic effects of the selected five process parameters were estimated. The analysis includes the coefficient of determination (R^2^) which determines the efficiency of the polynomial regression model, the predicted R^2^ value, the adjusted R^2^ value, the effect of each variable, Fisher test (*F* test) and probability *P *value. The Ni^2+^ regression model has R^2^ value = 0.9902, meaning that 99.02% (Table 2) of variations in the removal percentages of Ni^2+^ were due to the independent factors and the model cannot describe just 0.98% of the total variations. In addition, the methylene blue regression model has R^2^ value = 0.9944 (Table 3), meaning that 99.44% of the variations in the removal percentages of methylene blue were due to the independent factors and the model cannot describe just 0.56% of the total variations. A regression model with a high R-squared value greater than 0.9 is known to has a great, positive correlation^34^. On the other hand, the values of the adjusted determination coefficient (Adj R^2^) are 0.9835 and 0.9905 for Ni^2+^ and the methylene blue; respectively. The very high value of Adj R^2^ verified that the model was very significant (Tables 2, 3). The predicted R^2^ values of 0.9746 and 0.9840 for Ni^2+^ and the methylene blue removal %; respectively, were in an excellent agreement with the adjusted R^2^ values. That revealed a strong agreement between the predicted and observed values of Ni^2+^ and methylene blue removal %. The predicted and adjusted values of R^2^ must be within 20% of each other, so that we can say that there is a reasonable agreement between them^35^. Therefore, the models used in this study are optimal in the range of experimental variables in the prediction of methylene blue and Ni^2+^ removal %.

**Table 2:** Analysis of variance for biosorption of Ni^2+^ by *Gracilaria* seaweed biomass obtained by the Face-centered central composite design.

Source of variance	Sum of square	Degrees of freedom	Mean of square	*F*-value	*P*-value	Coefficient estimate
Model	4181.99	20	209.10	146.74	< 0.0001*	94.77
**Linear effect**						
X_1_	50.83	1	50.83	35.67	< 0.0001*	1.22
X_2_	252.86	1	252.86	177.46	< 0.0001*	2.73
X_3_	140.68	1	140.68	98.73	< 0.0001*	2.03
X_4_	208.84	1	208.84	146.56	< 0.0001*	2.48
X_5_	343.94	1	343.94	241.37	< 0.0001*	3.18
**Interaction effect**						
X_1_X_2_	25.84	1	25.84	18.13	0.0002*	− 0.90
X_1_X_3_	6.32	1	6.32	4.43	0.0440*	− 0.44
X_1_X_4_	1.80	1	1.80	1.26	0.2703	0.24
X_1_X_5_	1.50	1	1.50	1.05	0.3133	− 0.22
X_2_X_3_	100.96	1	100.96	70.85	< 0.0001*	− 1.78
X_2_X_4_	353.37	1	353.37	247.99	< 0.0001*	− 3.32
X_2_X_5_	124.01	1	124.01	87.03	< 0.0001*	− 1.97
X_3_X_4_	0.52	1	0.52	0.37	0.5495	− 0.13
X_3_X_5_	19.62	1	19.62	13.77	0.0009*	− 0.78
X_4_X_5_	2.06	1	2.06	1.45	0.2385	− 0.25
**Quadratic effect**						
X_1_^2^	111.07	1	111.07	77.95	< 0.0001*	− 6.70
X_2_^2^	23.98	1	23.98	16.83	0.0003*	− 3.11
X_3_^2^	16.17	1	16.17	11.35	0.0022	− 2.56
X_4_^2^	52.25	1	52.25	36.67	< 0.0001*	− 4.60
X_5_^2^	0.84	1	0.84	0.59	0.4479	0.58
**Error effect**						
Lack of fit	22.63	22	1.03	0.39	0.9590	
Pure error	18.69	7	2.67			

R^2^	0.9902	Std. Dev	1.19			
Adj R^2^	0.9835	Mean	83.63			
Pred R^2^	0.9746	C.V. %	1.43			
Adeq precision	53.44	PRESS	107.31			

*F* Fishers's function, *P* level of significance, *C.V* Coefficient of variation.

*Significant values.

**Table 3:** Analysis of variance for biosorption of methylene blue by *Gracilaria* seaweed biomass obtained by the Face-centered central composite design.

Source of variance	Sum of square	Degrees of freedom	Mean of square	*F*-value	*P*-value	Coefficient estimate
Model	3951.28	20	197.56	256.92	< 0.0001*	92.09
**Linear effect**						
X_1_	189.49	1	189.49	246.42	< 0.0001*	2.36
X_2_	295.96	1	295.96	384.88	< 0.0001*	2.95
X_3_	155.21	1	155.21	201.85	< 0.0001*	2.14
X_4_	487.87	1	487.87	634.44	< 0.0001*	3.79
X_5_	229.30	1	229.30	298.19	< 0.0001*	2.60
**Interaction effect**						
X_1_X_2_	25.21	1	25.21	32.79	< 0.0001*	− 0.89
X_1_X_3_	35.23	1	35.23	45.81	< 0.0001*	− 1.05
X_1_X_4_	3.58	1	3.58	4.66	0.0393*	0.33
X_1_X_5_	115.94	1	115.94	150.77	< 0.0001*	− 1.90
X_2_X_3_	35.24	1	35.24	45.83	< 0.0001*	− 1.05
X_2_X_4_	163.41	1	163.41	212.50	< 0.0001*	− 2.26
X_2_X_5_	39.79	1	39.79	51.75	< 0.0001*	− 1.12
X_3_X_4_	0.15	1	0.15	0.19	0.6639	− 0.07
X_3_X_5_	109.71	1	109.71	142.66	< 0.0001*	− 1.85
X_4_X_5_	4.98	1	4.98	6.48	0.0165*	− 0.39
**Quadratic effect**						
X_1_^2^	90.80	1	90.80	118.07	< 0.0001*	− 6.06
X_2_^2^	7.87	1	7.87	10.24	0.0033*	− 1.78
X_3_^2^	46.83	1	46.83	60.89	< 0.0001*	− 4.35
X_4_^2^	34.82	1	34.82	45.28	< 0.0001*	− 3.75
X_5_^2^	4.03	1	4.03	5.24	0.0296*	1.28
**Error effect**						
Lack of fit	17.14	22	0.78	1.06	0.5069	
Pure error	5.16	7	0.74			

R^2^	0.9944	Std. Dev	0.88			
Adj R^2^	0.9905	Mean	82.12			
Pred R^2^	0.9840	C.V. %	1.07			
Adeq precision	75.00	PRESS	63.64			

*F* Fishers's function, *P* level of significance, *C.V* Coefficient of variation.

*Significant values.

**Table 4:** Fit summary for Face-centered central composite design for biosorption of Ni^2+^ results.

Source	Sum of squares	*df*	Mean square	*F-*value	*P-*value *P*rob > *F*
**lack of fit tests**					
Linear	3207.47	37	86.69	32.46	< 0.0001*
2FI	2571.48	27	95.24	35.66	< 0.0001*
Quadratic	22.63	22	1.03	0.39	0.9590
**Sequential model sum of squares**					
Linear vs mean	997.15	5	199.43	2.72	0.0316*
2FI vs linear	635.99	10	63.60	0.83	0.5991
Quadratic vs 2FI	2548.85	5	509.77	357.75	< 0.0001*

Source	Standard deviation	R-squared	Adjusted R-squared	Predicted R-squared	PRESS
**Model summary statistics**					
Linear	8.56	0.2361	0.1493	0.0483	4019.20
2FI	8.73	0.3867	0.1161	− 0.2138	5126.30
Quadratic	1.19	0.9902	0.9835	0.9746	107.31

*df* degree of freedom, *PRESS* sum of squares of prediction error, *2FI* two factors interaction.

*Significant values.

**Table 5:** Fit summary for Face-centered central composite design for biosorption of methylene blue results.

Source	Sum of squares	*df*	Mean square	*F-*value	*P-*value *P*rob > *F*
**Lack of fit tests**					
Linear	2610.58	37	70.56	95.80	< 0.0001*
2FI	2077.35	27	76.94	104.46	< 0.0001*
Quadratic	17.14	22	0.78	1.06	0.5069
**Sequential model sum of squares**					
Linear vs mean	1357.84	5	271.57	4.57	0.0019*
2FI vs linear	533.24	10	53.32	0.87	0.5683
Quadratic vs 2FI	2060.20	5	412.04	535.83	< 0.0001*

Source	Standard deviation	R-squared	Adjusted R-squared	Predicted R-squared	PRESS
**Model summary statistics**					
Linear	7.71	0.3417	0.2669	0.1820	3250.22
2FI	7.83	0.4759	0.2447	− 0.0234	4066.50
Quadratic	0.88	0.9944	0.9905	0.9840	63.64

*df* degree of freedom, *PRESS* sum of squares of prediction error, *2FI* two factors interaction.

*Significant values.

The negative coefficient values suggest an antagonistic correlation among the variable(s) and the removal percentage while the positive coefficient implies a synergistic relationship between the variable(s) and the removal percentage. Consequently, the negative coefficients values of linear effects, mutual interactions effects between two variables and quadratic effects of the selected process parameters means that they exert a negative effect on methylene blue and Ni^2+^ removal % by the dry biomass of *Gracilaria* seaweed, whereas the positive coefficient values mean that they increase methylene blue and Ni^2+^ removal % by the dry biomass of *Gracilaria* seaweed in the tested range of the selected five process parameters. It can be seen from the coefficient values (Table 2) that the algal biomass concentration, initial pH level, nickel concentration and methylene blue concentrations and contact time had positive effects on Ni^2+^ removal %. However, all the variables (algal biomass concentration, initial pH level, nickel concentration, contact time and methylene blue concentration) had positive effects on the methylene blue removal % (Table 3).

In order to assess the significance of each parameter, *P*-values were used as a tool. In this case, the variables showing *P*-values below 0.05 were assumed to have significantly effects^36^. Analysis of variances (ANOVA) for a quadratic regression model of nickel removal (%) indicates that the model is highly important as is apparent from a very small probability value [*P-*value ˂ 0.0001] with the Fisher’s *F* test (*F-*value = 146.74) (Table 2). The *P*-values showed that linear coefficients for algal biomass concentration, initial pH level, Ni^2+^ concentration, methylene blue concentration and contact time were significant for nickel removal with probability values of < 0.0001 for all (Table 2). The interaction effects between X_1_X_2_ (*Gracilaria* seaweed biomass, initial pH), X_1_ X_3_ (*Gracilaria* seaweed biomass, initial Ni^2+^ concentrations), X_2_ X_3_ (initial pH, initial Ni^2+^ concentrations), X_2_X_4_ (initial pH, methylene blue concentrations), X_2_X_5_ (initial pH, contact time), X_3_ X_5_ (initial Ni^2+^ concentrations, contact time) are significant [*P-*value ˂ 0.05]. While, the interaction effects between X_1_ X_4_ (*Gracilaria* seaweed biomass, methylene blue concentrations), X_1_X_5_ (*Gracilaria* seaweed biomass, contact time), X_3_ X_4_ (initial Ni^2+^ concentrations, methylene blue concentrations), X_4_ X_5_ (methylene blue concentrations, contact time) are not significant [*P-*value ˃ 0.05]. Additionally, the coefficients *P*-values show that there are significant quadratic effects of algal biomass concentration, initial pH level, nickel concentration and methylene blue concentration. Among the five variables, the quadratic effect of contact time is not significant (Table 2).

On the other hand, the ANOVA of the quadratic regression model of methylene blue removal (%) indicates that the model is highly important as is apparent from a very small probability value [*P-*value ˂ 0.0001] with the Fisher’s *F* test (*F-*value = 256.92) (Table 3). All the variables (algal biomass concentration, initial pH level, nickel concentration, methylene blue concentration and contact time) were significant for removal of methylene blue with probability values of < 0.0001 (Table 3). The lower *P*-values denote that the factors are more significant on the methylene blue removal. The interaction effects between all the variables are significant except interactions between X_3_X_4_ (nickel concentration and methylene blue concentration) are not significant [*P-*value ˃ 0.05]. In addition, the *P*-values of the coefficients show that the quadratic effects of all the five variables are significant (Table 3).

Statistically analyzed data for nickel removal (%) indicates that the coefficient of variation percentage (C.V. = 1.43%) (Table 2) is substantially small, suggesting that the experiments carried out have a high degree of precision and reliability^37^. Adequate precision determines the level of noise; the level higher than 4 is preferable and implies the model reliability. The current adequate precision ratio of the Ni^2+^ removal model is 53.44, which means that the model was reliable. The predicted residual error sum of squares (PRESS) is an indication of how well every point is suitable for the design. The regression model PRESS value is 107.32. The model's mean and standard deviation are 83.63 and 1.19; respectively (Table 2). Meanwhile, statistically analyzed data for methylene blue removal (%) shows a comparatively low value of the coefficient of variation percentage (C.V. = 1.07%) (Table 3) confirms a high degree of experimental accuracy and reliability. The present methylene blue removal model had an acceptable, adequate precision ratio of 75.00 and this means the reliability of the model. The model's mean and standard deviation are 82.12 and 0.88; respectively. Value of PRESS for statistically analyzed data of methylene blue removal (%) is 63.64 (Table 3).

Tables 4 and 5 displays the fit summary results applied to the selection of the highest order polynomial model, which has the significant model terms and an insignificant lack of fit test. In addition, the model summary statistics indicate what model has higher adj. and pred. R^2^ and lower standard deviation. The fit summary results (Tables 4, 5) demonstrated that, the quadratic models of both nickel and methylene blue removal by the dry biomass of *Gracilaria* seaweed are very significant with a very low *P-*value < 0.0001. Lack of Fit Test for Ni^2+^ removal is non-significant (*P-*value = 0.9590) with *F*-value = 0.39. The model summary statistics for Ni^2+^ removal quadratic model (Table 4) recorded the lowest standard deviation of 1.19 and the highest adjusted and predicted R^2^ of 0.9835 and 0.9746; respectively. Furthermore, Lack of Fit Test for methylene blue removal is non-significant (*P-*value = 0.5069) with *F*-value = 1.06. The model summary statistics for methylene blue removal quadratic model (Table 5) recorded the smallest standard deviation of 0.88 and the highest adjusted and predicted R^2^ of 0.9905 and 0.9840; respectively.

The mathematical relationships between the chosen independent factors and the responses (nickel removal and methylene blue removal percentages) are given by the following polynomial equations of the second order:

1 $$\begin{aligned} & {\text{The predicted value of nickel removal }}\left( \% \right) = + \;94.77 + 1.22{\text{X}}_{1} + 2.73{\text{X}}_{2} + 2.03{\text{X}}_{3} + 2.48{\text{X}}_{4} \\ & + \;3.18{\text{X}}_{5} - 0.90{\text{X}}_{1} {\text{X}}_{2} - 0.44{\text{X}}_{1} {\text{X}}_{3} - 0.24{\text{X}}_{1} {\text{X}}_{4} - 0.22{\text{X}}_{1} {\text{X}}_{5} - 1.78{\text{X}}_{2} {\text{X}}_{3} {-}3.32{\text{X}}_{2} {\text{X}}_{4} \\ & - \;1.97{\text{X}}_{2} {\text{X}}_{5} - 0.13{\text{X}}_{3} {\text{X}}_{4} - 0.78{\text{X}}_{3} {\text{X}}_{5} - 0.25{\text{X}}_{4} {\text{X}}_{5} - 6.70{\text{X}}_{1}^{2} - 3.11{\text{X}}_{2}^{2} - 2.56{\text{X}}_{3}^{2} - 4.60{\text{X}}_{4}^{2} \\ & + \;0.58{\text{X}}_{5}^{2} \\ \end{aligned}$$

2 $$\begin{aligned} & {\text{The predicted value of methylene blue removal }}\left( \% \right) = + \;{92}.0{9} + {2}.{\text{36X}}_{{1}} + {2}.{\text{95X}}_{{2}} + {2}.{\text{14X}}_{{3}} \\ & + \;{3}.{\text{79X}}_{{4}} + {2}.{6}0{\text{X}}_{{5}} - 0.{\text{89X}}_{{1}} {\text{X}}_{{2}} - {1}.0{\text{5X}}_{{1}} {\text{X}}_{{3}} + 0.{\text{33X}}_{{1}} {\text{X}}_{{4}} - {1}.{9}0{\text{X}}_{{1}} {\text{X}}_{{5}} - {1}.0{\text{5X}}_{{2}} {\text{X}}_{{3}} \\ & - \;{2}.{\text{26X}}_{{2}} {\text{X}}_{{4}} - {1}.{\text{12X}}_{{2}} {\text{X}}_{{5}} - 0.0{\text{7X}}_{{3}} {\text{X}}_{{4}} - {1}.{\text{85X}}_{{3}} {\text{X}}_{{5}} - 0.{\text{39X}}_{{4}} {\text{X}}_{{5}} - {6}.0{\text{6X}}_{{1}}^{{2}} - {1}.{\text{78X}}_{{2}}^{{2}} - {4}.{\text{35X}}_{{3}}^{{2}} \\ & - \;{3}.{\text{75X}}_{{4}}^{{2}} + {1}.{\text{28X}}_{{5}}^{{2}} \\ \end{aligned}$$

where X_1_‒X_5_ are the coded values of *Gracilaria* seaweed concentration, initial pH value, nickel concentration, methylene blue concentration and contact time; respectively.

Above the second-order polynomial Eqs. (1), (2) indicate how the individual variables, their quadratic or their interactions among them affected the removal (%) of nickel or methylene blue from aqueous solution by using the biosorbent dry biomass of the *Gracilaria* marine algae. The negative coefficient values imply that the linear effects, quadratic effects or interaction effects between the variables have negative effects on the removal (%) of nickel or methylene blue (i.e. decreases the biosorption percentage), whereas positive coefficient values imply that the linear effects, quadratic effects or interaction effects between different factors increase the biosorption percentage of nickel or methylene blue in the tested ranges from the aqueous solutions.

### The normal probability plot (NPP) of the residuals

**Figure 2:**
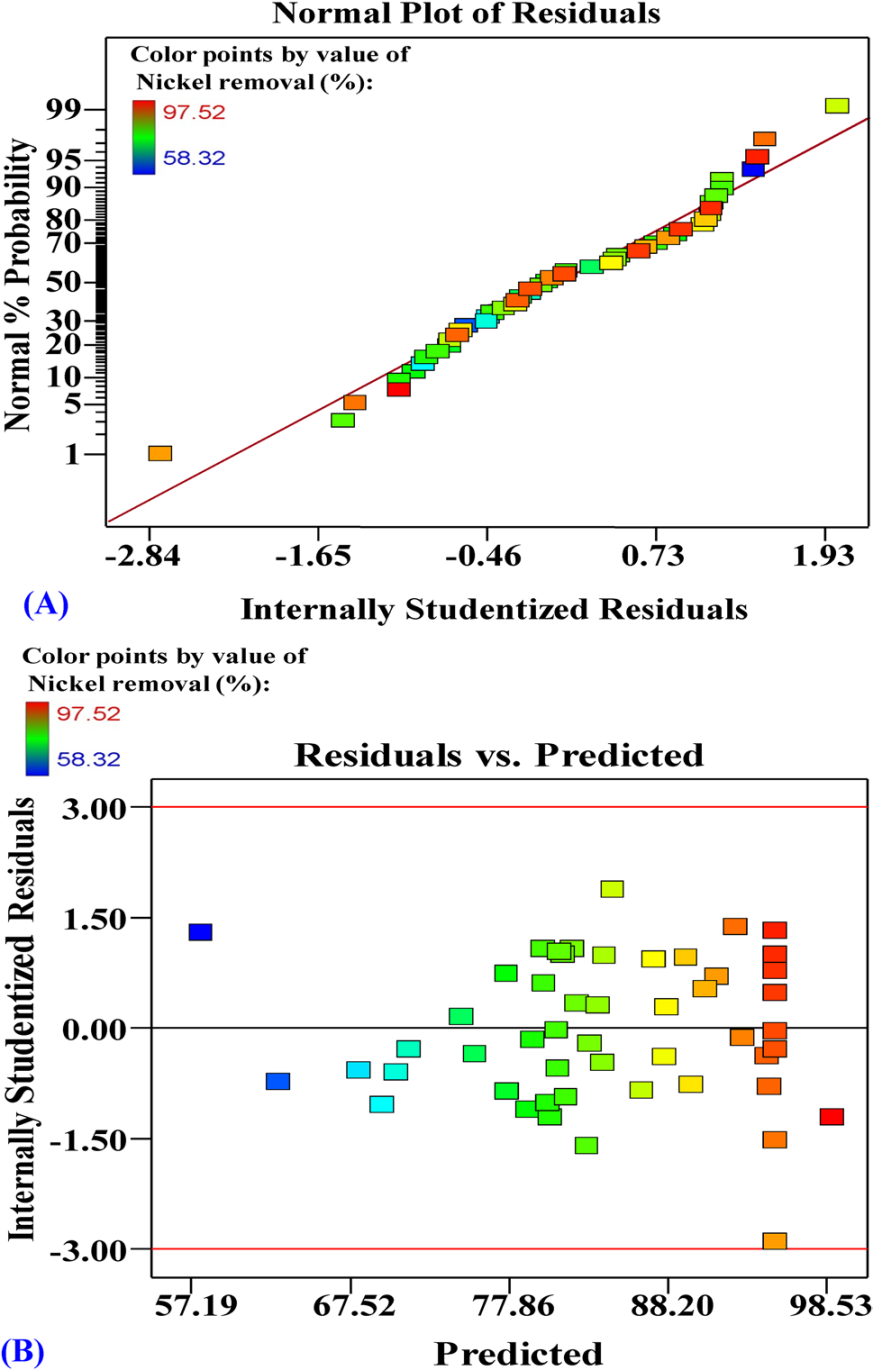
(**A**) Normal probability plot of internally studentized residuals, and (**B**) plot of internally studentized residuals versus predicted values of Ni^2+^ biosorption by *Gracilaria* seaweed biomass. This figure was created by using Design Expert version 7 for Windows software.

The NPP of the residuals is an essential statistical tool for verifying the suitability of the model^38^. Figure 2A shows the NPP of the residuals for Ni^2+^ removal (%) data analysis that indicating that the residuals distributed around the diagonal line of the normal distribution. This means that the model is adequate. Figure 2B displays a graph of predicted vs. residual values of the removal (%) of nickel. The graph indicates that the gathered points along the diagonal line showing the model's good fit.

### Effects of process variables on nickel and methylene blue biosorption (three dimensional surface plots)

The three-dimensional (3D) surface plots were generated in order to identify the optimum conditions for the bioprocess (removal of Ni^2+^ and methylene blue from aqueous solution) and to visualize the relationships between the responses and the interactions between the selected process parameters. 3D graphs for the five variables combined in pairs (algal biomass concentration, initial pH level, nickel concentration, methylene blue concentration and contact time) were created by plotting removal of Ni^2+^ (percent) on Z-axis versus two process parameters while other parameters maintained fixed at their midpoints.

**Figure 3:**
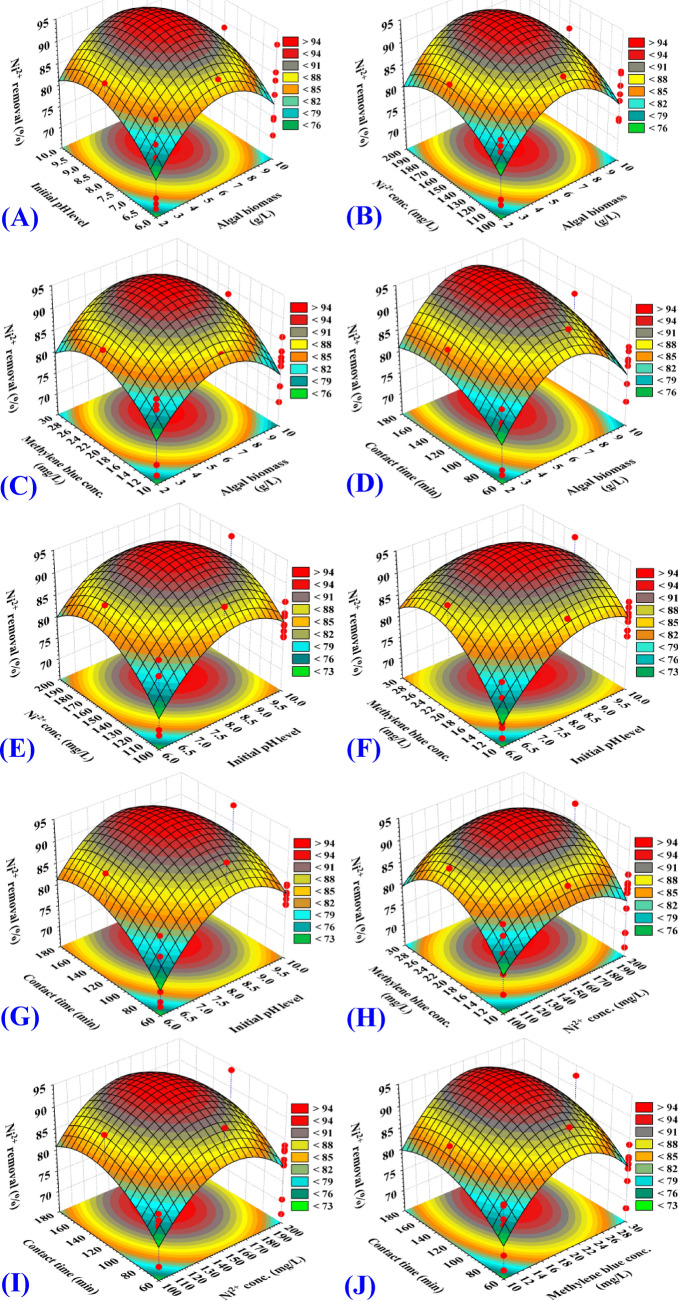
Three-dimensional surface plots for biosorption of Ni^2+^ by *Gracilaria* seaweed biomass (**A**–**J**), showing the interactive effects between the five tested variables. This figure was created by using statistical software package, STATISTICA software (Version 8.0, StatSoft Inc., Tulsa, USA).

**Figure 4:**
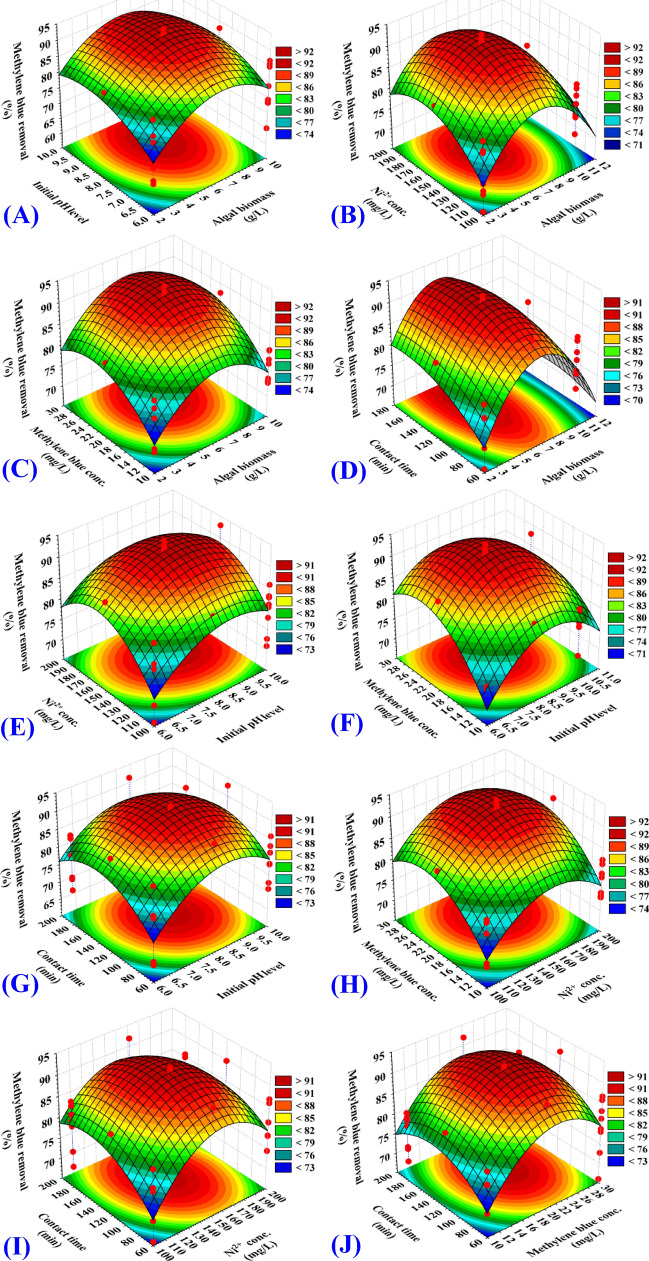
Three-dimensional surface plots of biosorption of methylene blue by *Gracilaria* seaweed biomass (**A**–**J**), showing the interactive effects between the five tested variables. This figure was created by using statistical software package, STATISTICA software (Version 8.0, StatSoft Inc., Tulsa, USA).

The three dimensional surface plots (3D) (Figs. 3A, 4A) shows the simultaneous effect of algal biomass concentration, (X_1_) and initial pH level (X_2_) on nickel removal (%) and methylene blue removal (%); respectively, while nickel concentration, methylene blue concentration and contact time (X_3_‒X_5_) were kept at their zero levels. The removal percentages of both nickel (Fig. 3A) and methylene blue (Fig. 4A) increases with the increase of initial concentration of algal biomass from 2 to 6 g/L. The increased removal percentages of nickel and methylene blue can be mainly due to the increased surface area and the availability of unsaturated adsorption binding sites on the surface of alga needed for the biosorption process reaction by increasing the algal biomass concentration. The percentage removal of nickel and methylene blue then decreased with increasing the algal biomass concentration from 6.5 to 10 g/L. At high algal biomass concentrations, the agglomeration of the biomass can be a reason for the decrease in the removal efficacy. An increase in the initial pH led to an increase in the percentage of removal up to pH 8 and then further increase in pH decreased the removal percentage. This means that the maximum removal percentages of nickel and methylene blue could be obtained by 6.4 g of the algal biomass and initial pH 8. This may be explained by the slightly alkaline pH 8 improved the access of nickel ions and methylene blue to the adsorption active sites of the algal biomass.

The three dimensional surface plots (3D) (Figs. 3B, 4B) shows the simultaneous effect of algal biomass concentration (X_1_) and nickel concentration (X_3_) on nickel removal (%) and methylene blue removal (%); respectively, while initial pH level, methylene blue concentration and contact time (X_2_, X_4_, X_5_) were kept at their zero levels. The removal percentages of both nickel (Fig. 3B) and methylene blue (Fig. 4B) increases with the increase of initial concentration of algal biomass. The percentage removal of nickel and methylene blue then decreased with increasing the algal biomass concentration from 6.5 to 10 g/L. An increase in the nickel concentration resulted in an increase in the nickel removal (%) up to 171 mg/L (by using 6.4 g/L of the algal biomass) and then further increase in the nickel concentration decreased the removal percentage (Fig. 3B). While, an increase in the nickel concentration up to 160 mg/L (by using 6.4 g/L of the algal biomass) resulted in an increase in the percentage removal of methylene blue and then further increase in the nickel concentration decreased the removal percentage (Fig. 4B).

The surface plots (Figs. 3C, 4C) shows removal (%) of nickel and methylene blue as a function of algal biomass concentration (X_1_) and methylene blue concentration (X_4_) while other parameters maintained fixed at their midpoints. By using high and low algal biomass concentrations, removal (%) of nickel and methylene blue decreased, suggesting that the removal was highly dependent on the concentration of algal biomass. The interaction effects between algal biomass concentration (X_1_) and methylene blue concentration (X_4_) are not significant (*P-*value = 0.2703) for removal (%) of nickel (Table 2). While, the interaction effects between algal biomass concentration (X_1_) and methylene blue concentration (X_4_) are significant for removal (%) of methylene blue (*P-*value = 0.0393) (Table 3).

Similarly, Figs. 3D and 4D shows that the percentages of removal of both nickel and methylene blue increased with an increase in the time of contact. Figures 3E and 4E with the increased concentration of nickel above 164 mg/L, the percentage of removals decreases. This may attributed to the saturation of binding sites on the surface of alga. Figures 3F and 4F shows that the percentages of removal of both nickel and methylene blue have increased with an increase in the initial pH level to pH 8 and then further increase in pH decreased the removal percentage. The effect of both initial pH level and contact time are also reflected in Figs. 3G and 4G. Similarly, the effect of both nickel and methylene blue concentrations are also reflected in Figs. 3H and 4H. The percentage removal of nickel increased with the increased concentration of nickel from 100 to 171 mg/L (Figs. 3H, 4H). In addition, a slight decrease in the removal percentage resulted from a further increase in the concentration of nickel. The high percentage removal of methylene blue was obtained using about 24.2 mg/L of methylene blue (Fig. 4H). In addition, the effects of contact time, nickel concentrations and methylene blue concentrations are also presented in Figs. 3I,J and 4I,J.

### Initial pH

Different variables can influence biosorption processes such as biosorbent dosage, contact time, initial metal concentration and pH. Among these variables, the initial pH level has been identified to be the most important variable that controls the pollutant biosorption process. The pH levels influences the activity of the functional groups of the biomass, the net charge on the biosorbent cell surface as well as affect the chemistry of metals solutions^39^. The biosorption process of heavy metals is highly pH-dependent, may be minimized or increased by competition between heavy metal ions and H^+^ at the same cellular active sites on the biosorbent cell surface^40^.

Experimental results in this study clearly indicate that the rise in pH enhances the biosorption of nickel by the red algae *Gracilaria* biomass, and reaches the maximum at pH 8, however; a very high pH increase can lead to nickel precipitation that must be avoided^41^. pH affects the stability and state of nickel species [Ni^2+^, insoluble nickel(II) hydroxides at different pH values] in aqueous environments, as well as the properties of the adsorbent surface^5^. In acid, neutral, and fairly basic solutions, Ni^2+^ is the predominant form. When pH is > 8, precipitation of insoluble nickel(II) hydroxides occurs^5^. In this study, the strong pH dependence of nickel biosorption could be attributed to the strong electrostatic attraction at higher pH taking place between the *Gracilaria* seaweed biomass and nickel ions.

In addition, the influence of pH on the biosorption process of metal ions could be clarified by the association–dissociation of many functional groups that have existed on the algal biomass surface, like hydroxyl and carboxyl groups. Since there is an excess of H_3_O^+^ and H^+^ at low pH, carboxylic groups are acidic and are not dissociated in the solutions (existing in a protonated state). As a result, the repulsive forces between these protonated carboxylic groups and heavy metal ions (positively charged) result in lower biosorption capacity at low pH levels^31,42^. At low pH, high concentrations of H^+^ ions in solution compete with nickel ions for available functional groups on the adsorbent, consequently reducing the effective exchange of metal ions (Ni(II) ions) on the sorbent surface as the sorbent surface takes up more H^+5^. However, the concentration of the proton in solution is lower than that of the metal ions with a further rise in the pH level, and the surface of the sorbent accumulates more negative charges that attract more metal ions. This facilitates the electrostatic attraction of positively charged Ni(II) ions toward fairly negatively charged adsorbent surface^43^. At higher pH levels (pH 8) deprotonation of functional groups with negative charges such as hydroxyl, amine and carboxyl on the surface of the sorbent increases the electrostatic attraction of positively charged heavy metal ions^31,44^ and improves the metal biosorption^5^.

At higher pH levels (more than pH 8), the decrease in the biosorption efficiency may be correlated with the repulsion between anionic solution negative charges and the negative charges of the biosorbents' surface^45^. Precipitation of insoluble anionic hydroxide complexes (Ni(OH)_2_) occurs at the high increase in pH (pH > 8.3) that reduces the free metal ions concentration and decrease the biosorption capacity for metals^31,42,44^. Vakili et al.^5^ reported that acidic pH is not favorable for the adsorption of nickel ions and maximum sorption capacities were obtained at pH ranging from 6 to 8.

Biosorption of methylene blue was done by using the red algae *Gracilaria* as an biosorbent. Experimental results in this study clearly indicate that the rise in pH enhances the biosorption of methylene blue dye by the red algae *Gracilaria* biomass, and reaches the maximum biosorption at around pH 8. The result can be explained by the protonation and de-protonating of the methylene blue dye as well as the red algae *Gracilaria* surface as biosorbent. The ionic attraction regulates the adsorption of the dye on the surface of biomass^46^. At acidic pH values, the surface of the biosorbent carries a positive charge due to the presence of H^+^ in the solution. This leads to electrostatic repulsion between the cationic methylene blue dye and positively charged *Gracilaria* surface as biosorbent. On the contrary, at alkaline pH values, due to the existing of OH^−^ ions in excess, the surface of the biosorbent carries a negative charge. This results in electrostatic attraction between cationic methylene blue dye and negatively charged *Gracilaria* surface leading to enhanced adsorption at higher pH values. Hammud et al.^47^ reported that the maximum MB removal by *Carolina* sp. was attained at pH 6.8. While, Khaled et al.^48^ recorded the maximum MB removal by *Ulva lactuca* at pH 8. On the other hand, Tahir et al.^12^ obtained maximum MB removal at pH 7 with *Sargassum*s pecies and *Ulva lactuca*. Rubin et al.^49^ also found maximum removal of MB at pH 7 using *Sargassum muticum*. However, Vilar et al.^50^ recorded that MB removal using *Gelidium* biomass based material was unaffected in the pH range from 4 to10 but was impaired at low pH.

The biosorption of the colored dye cations on the surface of the adsorbent has been mainly affected by the charges of the algal cell walls, which in turn were affected by the solution pH^51^. The algal cell walls, mainly made up of carbohydrates and protein, have functional groups such as sulfate, carboxyl, hydroxyl, amino, etc. that can serve as metal binding sites^52^. The maximum adsorption percentage of methylene blue removal value was 92% from solutions using marine algae *Ulva lactuca* was found at around pH 8^53^. A fewer anionic adsorption sites were generated on the surface of the biosorbent (the dried algal biomass) at low pH values, and the sorption process decreased probably due to electrostatic repulsion between excess H^+^ in the solution and the cationic dye molecules for sorption sites on the dried algal biomass^51^.

### Contact time

The time of contact influences the efficiency of the biosorption process. As the time of contact increases, the removal percentage increases as well. In the present study, experimental results clearly indicate that the percentage of simultaneous removal of Ni(II) ions and methylene blue by the red algae *Gracilaria* as biosorbent increases as the contact time increase up to the optimum, and reaches the maximum at 177.88 min. The increase in the rate of removal might be because of the availability of vacant functional groups on the biosorbent surface. Further increase in contact time leads to the occupation of active sites which results in the saturation of the algal cell surface causing an equilibrium state and no additional adsorption^54^.

In the case of different forms of biosorbents, the optimal contact time for maximum removal percentage is varied. Ibrahim^42^ reported that the optimum contact time for red macroalgae was 60 min. While, Ahmad et al.^31^ reported that the optimum contact time for the immobilized algal mass was 300 min (240 min for free suspended mass). Akhtar et al.^4^ found that adsorption of nickel onto the biomass of *Chlorella sorokiniana* immobilized in loofa sponge was rapid and reached equilibrium after only 15 min. On the contrary, the biosorption capacity increased with increasing contact time up to 110 min, after which the equilibrium was attained and the biosorption capacity is less constant^53^. Pahlavanzadeh et al.^39^ stated that the biosorption capacity of nickel by dried algal biomass increased with increased time of contact and the equilibrium was attained within 120 min. Xu et al.^55^ reported that adsorption attained equilibrium after 168 h (7 days). Obviously, such variability of the equilibrium time depends on the used adsorbent. This difference could be related to their structural and physicochemical characteristics diversity (e.g. surface area, pore volume, pore size, permeability, and density and availability of adsorption sites)^56^. Materials with higher adsorption capacity needs a longer time to reach equilibrium, implying that the advantage of a larger capacity is counter balanced by a longer treatment time, and vice versa^5^.

### Initial Ni(II) and methylene blue concentrations

The initial concentrations of metal ions and methylene blue also affect the biosorption process. As a general, increasing the initial concentrations of metal and dye usually leads to an increase in the percentage of removal as it offers a driving force that causes metal ions and dye to pass onto the surface of the biosorbent particles. An increase in the initial concentration of nickel ions leads to an increase in the sorption capacities of nickel ions on the surface of adsorbents^5^.

### Algal biomass concentration

The availability and low cost are the key reasons for selecting biomass for large-scale industrial uses^57^. Algae are known as among the most promising biosorbents types. Algal biomass has a wide range of advantages of removing heavy metals from wastewater, such as cost-effective renewable natural biomass, high metal removal efficiency, high absorption capacity, biomass regeneration and re-uses^58^. Large-scale biosorption using dead biomass can be easier and more applicable than living microorganisms, since the latter require a supply of nutrients^59^. The effect of metabolic processes of living microorganisms on biosorption is often not appreciated.

The increase in simultaneous biosorption capacity of methylene blue and nickel with an increase of the biosorbent concentration (red algae *Gracilaria*) is mostly attributed to the accessibility of many more unoccupied active sites on the surface of biosorbent^60^. The biosorption capacity of the surface of the biosorbent is specified by the availability of the active sites, for example, phosphate, imidazole, sulfate, sulphuryl, phosphoryl, amine, hydroxyl, carboxyl, etc.^61^. However, decreased biosorption capacity at higher concentrations of *Gracilaria* biomass could be attributed to the agglomeration of *Gracilaria* biomass, which in turn could reduce the intercellular spacing leading to a reduction of the total effective surface area available for the biosorption process and consequently decrease the number of active binding sites on the algal biomass surface available to simultaneous removal of dye and metal ions^51,60,62^.

However, Kavitha et al.^63^ stated that the decrease in biosorption process efficiency with the higher biosorbent dose can be attributed to blocking the binding sites from metal ions. Hannachi et al.^23^ reported the biosorption potential of *G. verrucosa* for Zn^2+^ ions removal. They found that the biosorption percentage of Zn (II) increases with an increase in the initial concentration of *G. verrucosa* biomass from 1 to 4 g/L and there is no substantial increase in the biosorption percentage of Zn(II) when the concentration of *G. verrucosa* biomass increases beyond 4 g/L. This has been explained by the increased number of unsaturated active adsorption sites on the biosorbent surface at a high concentration of algal biomass, and the metal ions available in the solution are insufficient to bind to all available binding sites, resulting in a low percentage of metal biosorption^23^.

On the other hand, Vijayaraghavan et al.^64^ observed that the biosorption percentage of methylene blue (MB) from aqueous solution increased with increase in *Gracilaria corticata* biomass concentration up to 5 g/L and further increase in the concentration of *Gracilaria corticata* biomass had no impact on the removal of percentages. The biosorption percentage of the dye decreases by increasing the concentration of the algal biomass and this has been explained by the lack of sufficient dye molecules at high sorbent dosages to cover all the active adsorption sites on the algal biomass, resulting in low dye biosorption percentage^64^. On the contrary, Arumugam et al.^65^ stated that the metal ion removal is proportional to the increasing seaweed dosage from 2 to 10 g. They suggested that a higher concentration of the algal biomass provides higher surface area and more adsorption active sites to bind to a constant concentration of metal ions. Therefore, more metal ions bind to the surface of seaweed and increase the removal percentage.

Numerous studies have been conducted on the adsorption of single-pollutant, despite the fact that large quantities of both dyes and metals are present together in the industrial wastewaters. High priority was therefore given to the simultaneous removal of dyes and heavy metal ions during wastewater treatment processes. Biosorption is a method used to treat wastewater efficiently by removing toxic metal ions and dyes from contaminated effluents using cost-effective and efficient natural biosorbents. Marine algae (seaweeds) are sustainable natural biomass, have been shown to be highly effective biosorbents with a high binding ability to a wide range of metals and dyes contaminants in aqueous effluents. Dried biomass of marine algae have proven to be more favorable than living cells as biosorbents because it does not require nutrient supply, it can be preserved and used prolonged periods at room temperature and can be regenerated and reused for multiple treatment cycles. Biosorption process has been affected by many environmental factors. Optimization of the operating process variables is crucial to achieving maximum biosorption. Response surface methodology is a mathematical and statistical technique used over several decades to optimize different process variables. It is very advantageous than conventional single variable optimization method as it helps to determine the optimal levels of the variables and helps to explore the interaction effects between various process factors in order to achieve maximum response.

### The model adequacy

**Figure 5:**
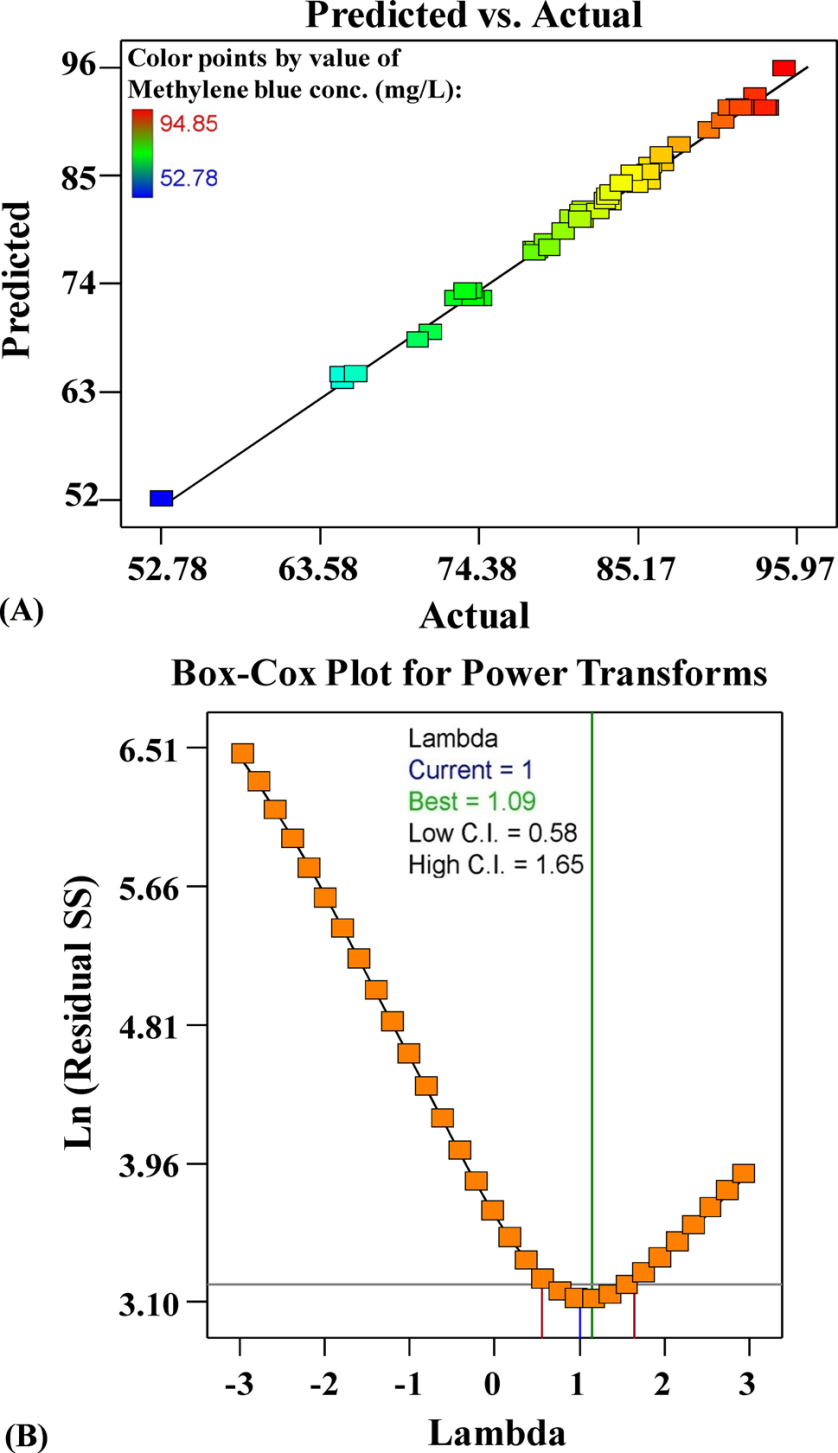
(**A**) Plot of predicted versus actual, and (**B**) Box–Cox plot of model transformation of methylene blue biosorption by *Gracilaria* seaweed biomass. This figure was created by using Design Expert version 7 for Windows software.

In the application of regression models, the main goal is to assess the adequacy of the model to predict the response variable. Figure 5A shows the actual versus predicted percentages for a simultaneous removal percentage of methylene blue from aqueous solution by *Gracilaria* seaweed biomass. Figure 5A displays all the points along the diagonal line, indicating that the model’s predicted percentages coincide with the actual percentages, confirming that the model is accurate. Figure 5B shows Box–Cox plot of model transformation has generated for a simultaneous removal percentage of methylene blue from aqueous solution by *Gracilaria* seaweed biomass. Box–Cox plot of model transformation method can help checks data that are not normally distributed by transforming the data for normalization. As can be seen in Fig. 5B, the optimal value of Lambda (λ) of 1 is lies between the two vertical red lines so that data transformation is not required.

### Desirability function (DF)

**Figure 6:**
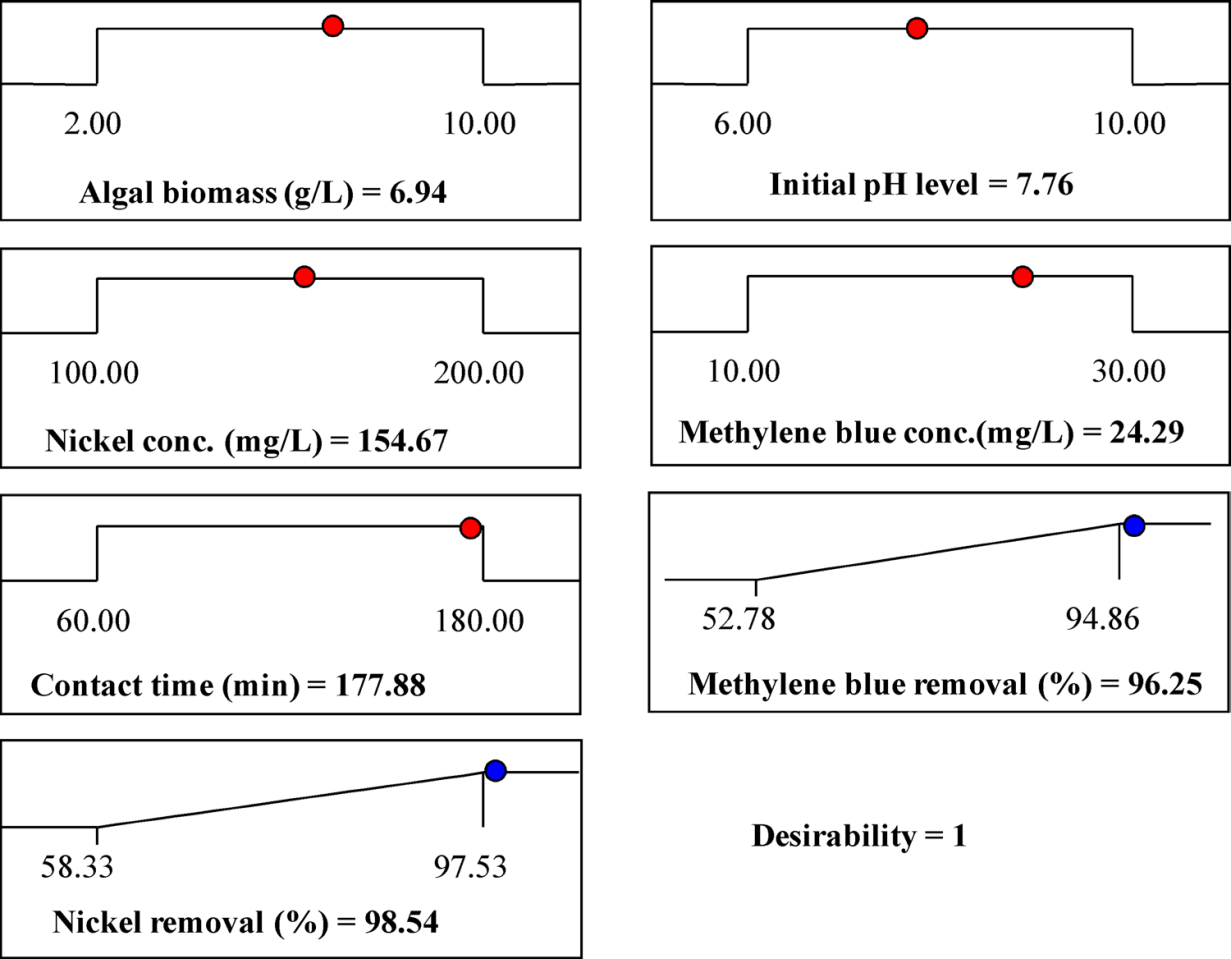
The optimization plot displays the desirability function and the optimum predicted values for the maximum simultaneous removal percentage of nickel ions and methylene blue.

The main goal of the experimental design is to determine the optimal predicted conditions for maximizing the responses. The desirability function (DF) was used to find the optimal predicted conditions for maximum response^66^. The values for DF varied from zero (undesirable) to one (desirable). The numerical optimization finds the points that maximize the desirability function. The DF option in the Design Expert Software (version 7.0.0) was used for the optimization process. Figure 6 shows the optimization plot displays the desirability function and the optimum predicted values for the maximum simultaneous removal percentage of methylene blue and nickel ions. The optimal predicted conditions attained using the desirability function for the maximum simultaneous removal percentages of the nickel ions and methylene blue were the algal biomass concentration of 6.94, initial pH level of 7.76, initial nickel ions concentration of 154.67 mg/L, initial concentration of methylene blue of 24.29 mg/L and contact time of 177.88 min. The conditions could be resulted in the removal percentages of 98.54% and 96.25% (with DF of 1) for nickel ions and methylene blue; respectively.

In order to verify the removal percentages of the nickel ions and methylene blue under the optimal predicted conditions, the experiments have been done in triplicate and compared with the predicted values. The experimental removal percentages averages of nickel ions and methylene blue were 98.9% and 97.31; respectively. The verification showed that the observed and predicted values of a strong agreement imply that the DF effectively determines the optimal predicted conditions for the simultaneous removal of nickel ions and methylene blue.

### Surface morphology

**Figure 7:**
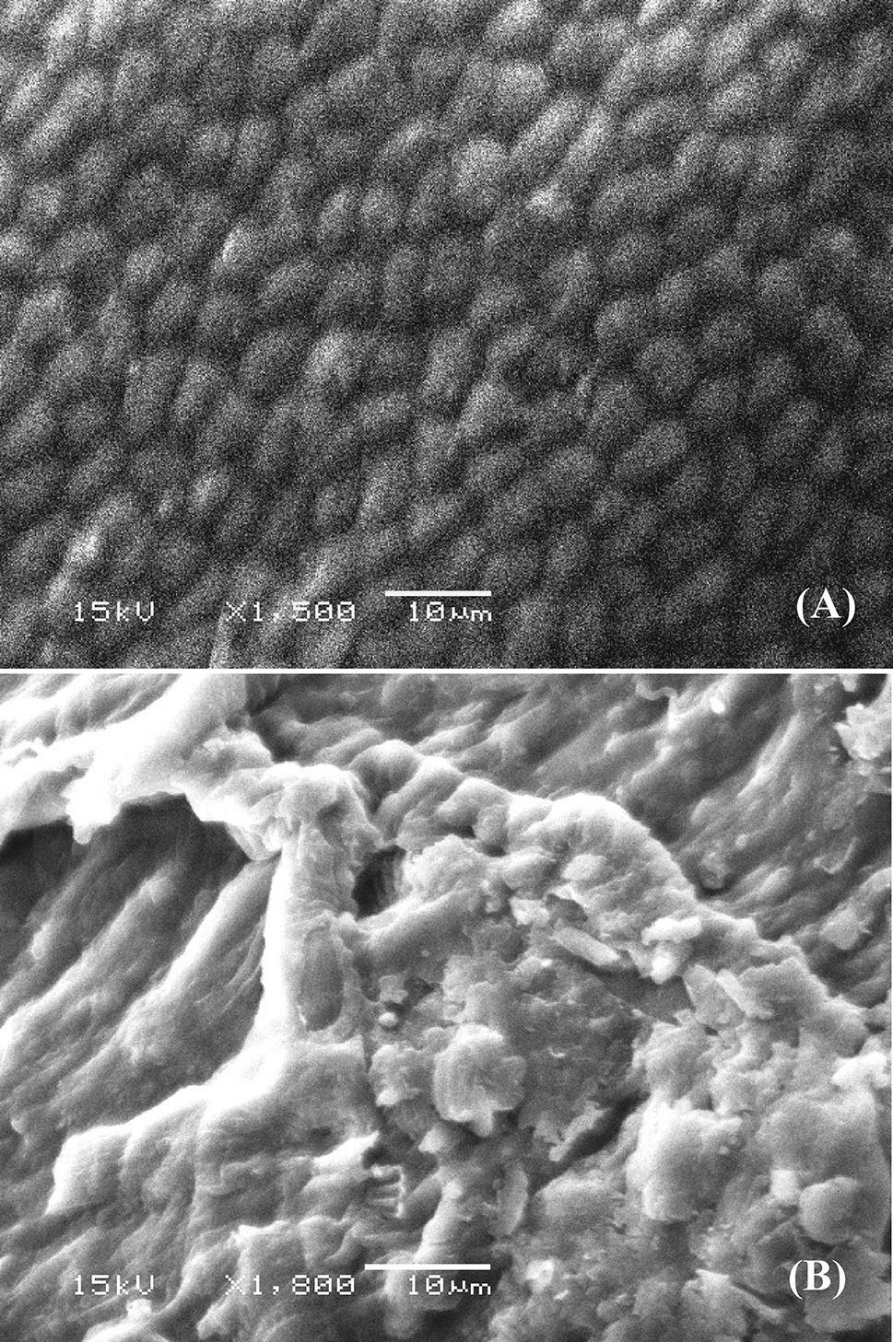
SEM micrograph of *Gracilaria* seaweed biomass: (**A**) before and (**B**) after simultaneous biosorption of methylene blue and Ni^2+^.

The surface morphology of *Gracilaria* seaweed biomass was analyzed by scanning electron microscopy before and after the biosorption process. The micrograph before the biosorption process revealed a regular surface of the biosorbent (Fig. 7A). After biosorption of methylene blue and nickel ions, the surface of the biosorbent was filled with adsorbate ions. Figure 7B clearly shows the presence of new glossy massive particles and layers on the surface of the biosorbent which are absent on the surface of the biosorbent before the biosorption process. Obvious morphological changes were seen in the cell surfaces (Fig. 7B), such as shrinking of surface. These changes may be due to strong crosslinking between nickel ions and negatively charged functional groups on the surface of *Gracilaria* seaweed biomass.

### FTIR spectra analysis

**Figure 8:**
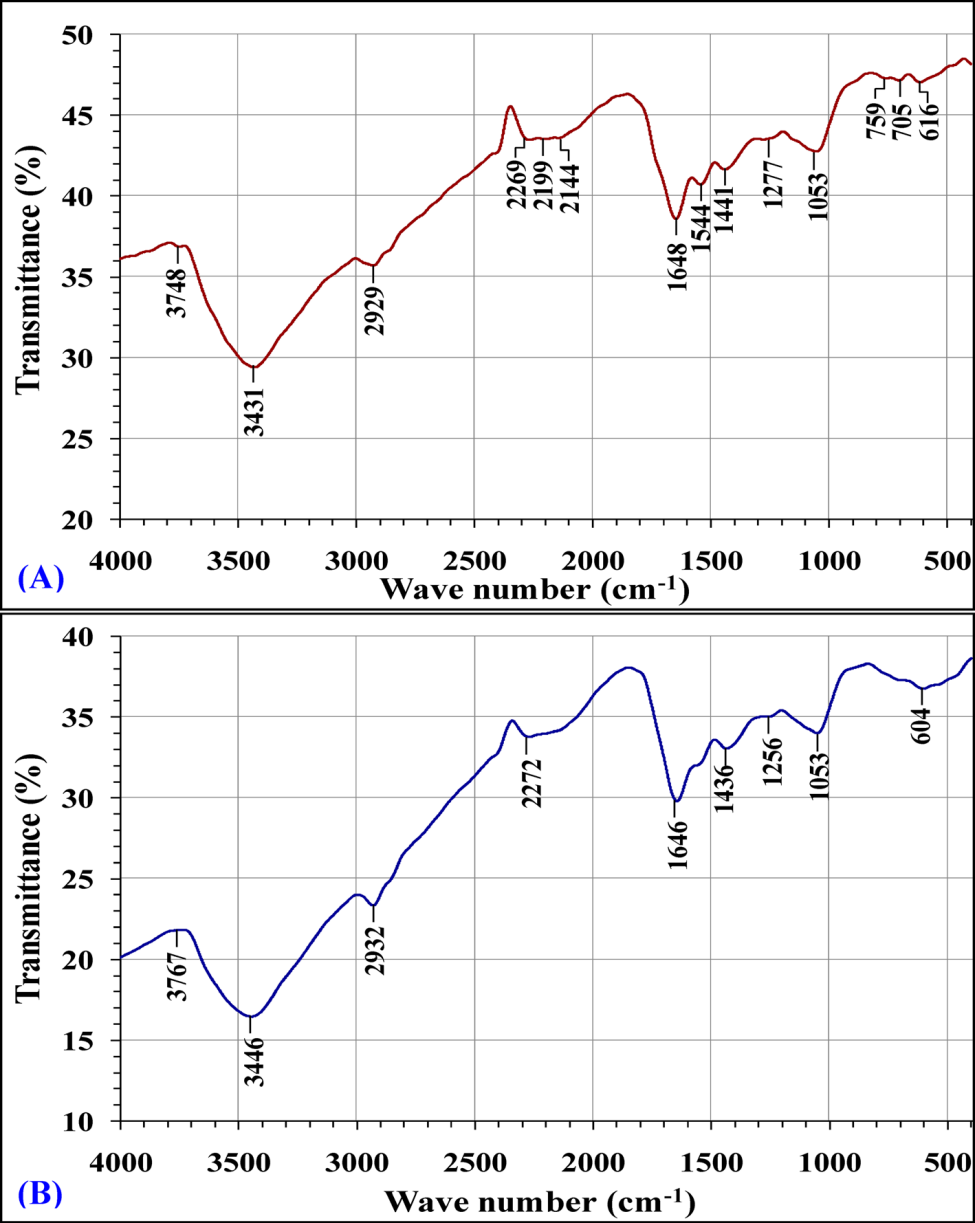
FTIR analysis of *Gracilaria* seaweed biomass: (**A**) before and (**B**) after simultaneous biosorption of methylene blue and Ni^2+^.

The FTIR spectra of *Gracilaria* seaweed biomass samples were analyzed before and after simultaneous bioremoval of methylene blue and nickel ions (Fig. 8 and Table 6) to detect any variation in the morphological features and surface characteristics due to the interaction of methylene blue and nickel ions with the functional groups. Biosorption of metal ions occurs via an ion exchange process on the cell surface. FTIR spectroscopy was often used to detect changes in the frequency of vibration in seaweeds^67^. The extent of band shift reflects the degree to which functional groups interact with the adsorbed ions^67^.

**Table 6:** FTIR spectral analysis of *Gracilaria* seaweed biomass before and after biosorption of methylene blue and nickel ions.

Before biosorption		After biosorption		Shift	References
Wave no. (cm^−1^)	Annotations	Wave no. (cm^−1^)	Annotations		
3748	Free Si–OH	3767	–OH hydroxyls groups	19	Cornac and Janin^68^
3431	–OH stretching	3446	–OH, N–H stretching	15	Ibitoye et al*.*^69^, Ramakrishna et al*.*^70^
2929	C–H stretching	2932	C–H stretching	3.86	Toteva et al*.*^72^, Raskó and Kiss^71^
2269	N=C=O groups (isocyanate)	2272	–O–C≡N groups	3	Júnior et al.^73^
2199	C≡N stretching (nitrile group)			72	Ruhland et al.^74^
2144	C–H stretching in alkanes or alkyne C≡C triple bond stretch			128	Coelho et al.^75^; Zhang et al.^76^; Mamunya and Iurzhenko^77^
1648	C=O, C=C stretching	1646	C=O, C=C stretching (amide-I, protein C=O stretching)	2	Shashoua et al*.*^78^, Sławomir^79^
1544	C=C stretching or Amide–II (protein N–H bend, C–N stretch)	–	–	–	Ivanova and Kolev^81^, Mallappa and Muniswamy^82^
1441	CH_2_–bending vibrations of the methyl group; lipids and proteins)	1436	O–CH_3_ methyl esters	–5	Drasar and Khripach^83^; Singh et al.^57^
1277	C–O stretching	1256	PO asymmetric vibrations (phosphate I)	–21	Ashtarinezhad et al*.*^84^, Sławomir^79^
1053	PO_4_ group vibration	1053	PO_4_^3−^ group vibration	–	Dekhili et al.^85^
759 705	C–S stretching	–	–	–	Panel et al.^86^
616	Phosphate	604	Phosphate	–11	Vivekanandhan et al*.*^87^, Hussein et al.^88^

The spectra of *Gracilaria* seaweed biomass were measured within wave number range from 400 to 4000 cm^−1^. FTIR spectrum of dry *Gracilaria* seaweed biomass sample before simultaneous bioremoval of methylene blue and nickel ions showed the characteristic absorption peaks at 3748, 3431, 2929, 2269, 2199, 2144, 1648, 1544, 1441, 1277, 1053, 759, 705 and 616 cm^−1^. FTIR spectrum of dry *Gracilaria* seaweed biomass sample after simultaneous bioremoval of methylene blue and nickel ions showed the previously mentioned characteristic signals were shifted to 3767, 3446, 2932, 2272, 1646, 1436, 1256, 1053 and 604 cm^−1^. Significant shifts in the absorption peaks after the biosorption confirm the significant role of the functional groups on *Gracilaria* biomass surface in the biosorption of Ni^2+^ and methylene blue dye from aqueous solution.

In the FTIR spectrum of the dry *Gracilaria* seaweed biomass sample before biosorption of methylene blue and nickel ions, the characteristic absorption peak at 3748 cm^−1^ is attributed to vibration of free Si–OH groups^68^ shifted after the biosorption process to peak at 3767 cm^−1^ which assigned to –OH, N–H stretching. The peak at 3431 cm^−1^ (before biosorption) represents –OH stretching vibrations^69^ shifted to 3446 cm^−1^ (after biosorption) which assigned to –OH or N–H stretching vibrations^70^. The peak at 2929 cm^−1^ is assigned to C–H stretching^71^ shifted after the biosorption process to peak at 2932 cm^−1^ which assigned to C–H stretching^72^. Whereas, Júnior et al.^73^ recorded that the signal at 2269 cm^–1^ corresponding to the N=C=O (isocyanate) groups. It is found that the signal at 2199 cm^−1^ was assigned to the nitrile group (C≡N)^74^. However, peak at 2144 cm^−1^, assigned to the vibrations of hydrocarbons, alkynes (C≡C) non terminal groups^75^. Significantly, the signal at 2144 cm^-1^ can be assigned to the C-H stretching in alkanes or triple bond stretch of alkyne C≡C^76^. The three peaks at 2269, 2199 and 2144 cm^−1^ are transformed into a single peak at 2272 cm^−1^ (after biosorption process) which is belonging to –O–C≡N groups (free isocyanate groups)^77^. The major peak shift suggests that the nitrile, alkanes, alkynes and isocyanate groups participated in the process of biosorption. The peak at 1648 cm^−1^ in the spectral range (before biosorption) is characteristic for the C=O, C=C stretching^78^ shifted to the peak at 1646 cm^−1^ in the spectral range (after biosorption) is characteristic for the carbonyl group stretching (C=O) or C=C stretching (amide –I, protein C=O stretching) or carboxyl (–CO) groups^23,79^. Stretching vibrations in the range of 1400 to 1657 cm^–1^ are assigned to the vibrations of N–H and C=O bonds of the protein amide group^79^. The FTIR peaks located at 1640 and 1540 cm^−1^ indicate the presence of proteins^80^. In addition to these peaks, one peak at 1544 cm^−1^ in the spectral range may be attributed to stretching vibration of carboxyl (–CO), C=C stretching or amide-II (C–N stretch, protein N–H bend) groups^23,81,82^, this peak disappeared in the FTIR spectrum of the *Gracilaria* biomass sample after biosorption process. The peaks at 1530–1560 cm^–1^ are attributed to stretching vibration of amino groups (NH stretching)^67^.

In parallel, the CH_2_-bending vibrations of the methyl group in lipids and proteins peak at 1441 cm^−1^^83^ is shifted to lower wave number at 1436 cm^−1^ of the methyl peak (O–CH_3_ methyl esters)^57^, that indicated the involvement of the methyl group in the biosorption process via ion exchange. The peak at 1277 cm^−1^ was attributed to stretching of C–O stretching vibrations shifted to peak at 1256 cm^−1^ (–21) which attributed to stretching vibration of the PO^2−^ asymmetric vibrations (phosphate I)^79,84^. The signal at 1053 cm^−1^ was attributed to the PO_4_^3−^ group vibration^85^ and not shifted after the biosorption process. The presence of intense peaks in the region of 1000–1100 cm^−1^ is a characteristic of polysaccharides^80^. Additionally, two peaks have appeared in the FTIR spectrum of the *Gracilaria* biomass sample before the biosorption process of methylene blue and nickel ions at 759 and 705 cm^−1^ which attributed to the stretching vibration of C–S^86^. These peaks disappeared in the FTIR spectrum of the *Gracilaria* biomass sample after biosorption process. *Gracilaria* species are known to produce agar with relatively high sulfate content^80^. Whereas, the signal at 616 cm^−1^ (before biosorption) shifted to peak at 604 cm^−1^ (after biosorption) which represents the phosphate groups^87,88^.

These shifts in the absorption peaks confirm the association of these functional groups on *Gracilaria* biomass in the biosorption process of methylene blue and nickel ions. Finally, the existing FITR spectra indicated that the key groups participating in the biosorption of methylene blue and nickel ions were methyl, alkynes, amide, phenolic, carbonyl, nitrile and phosphate groups.

## Materials and methods

### Preparation of the biosorbent

The red alga, *G. verrucosa*, used in this study, which classified under Rhodophyta, was harvested from the Mediterranean Sea coast of Abu-Qir, Alexandria, Egypt during summer season, 2019. Algal biomass was thoroughly washed with running tap water to remove salts, sand and any other external particles that may be attached to the surface. Algal biomass was finally washed with distilled water, immediately after the running tap water. The washed algal biomass was then air dried to a constant weight was obtained. The dried algal biomass was crushed and the particles with an average size of 1‒1.5 mm were selected and stored until used for biosorption experiments.

### Preparation of nickel and methylene blue dye solutions

Nickel standard solution was prepared by the precise dissolving of weighted nickel (II) sulfate (NiSO_4_) in distilled water at a concentration of 1000 mg/L. The desired work concentrations were then prepared by dilution of nickel stock solution.

The methylene blue stock solution was prepared by the precise dissolving of weighed dye in distilled water at a concentration of 1000 mg/L. The desired work concentrations were then prepared by dilution of the methylene blue stock solution.

### The biosorption experiments

**Figure 9:**
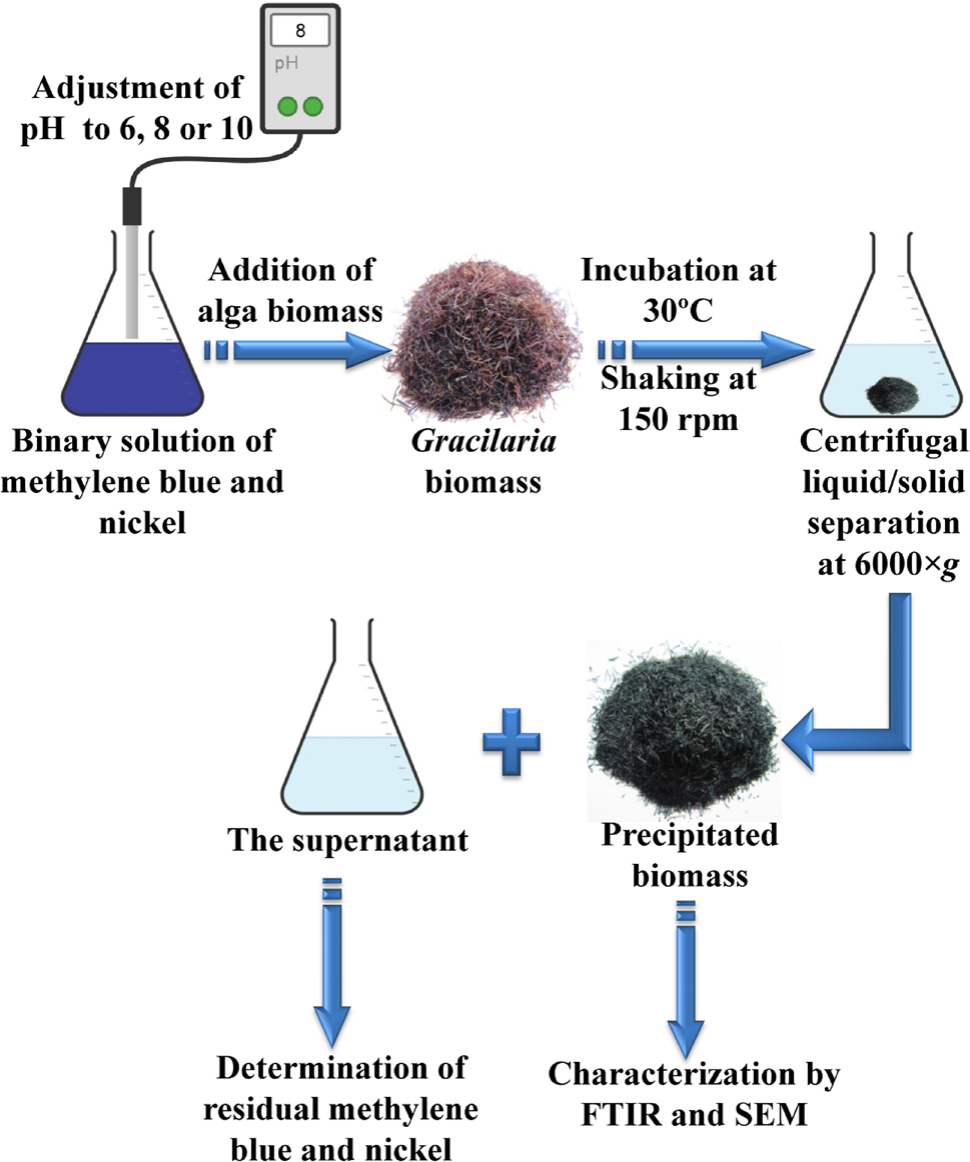
Schematic illustration of batch experimental biosorption steps to assess the efficiency of *Gracilaria* seaweed biomass for simultaneous bioremoval of methylene blue and nickel ions from aqueous solution.

The biosorption experiments were conducted in in batch mode in 250 mL flasks with 100 mL working volume to evaluate the efficiency of *Gracilaria* seaweed biomass for simultaneous bioremoval of methylene blue and nickel ions from aqueous solution (schematically illustrated in Fig. 9). Dry *Gracilaria* seaweed biomass was thoroughly mixed with the previously prepared solutions of methylene blue and nickel with different concentrations as demonstrated in the 50 trials (Table 1). The initial pH has been adjusted with the addition of 0.1 N NaOH or 0.1 N HCl to each solution. The suspensions were incubated and agitated at 150 rpm in a shaker incubator at 30 °C for period contact time.

### Optimization of biosorption experiments for simultaneous bioremoval of methylene blue and nickel by Face-centered central composite design (FCCCD)

Five-variables FCCCD with eight center runs was generated in this study with Design Expert Software (version 7.0.0). FCCCD was used to determine the optimum level of each variable to achieve the maximum efficiency for simultaneous bioremoval of methylene blue and nickel ions from aqueous solution. In addition, help to explore the linear, quadratic and interactions effects between the selected process parameters that have a significant impact on the bioremoval of methylene blue and nickel ions. The five variables used were as follows: initial Ni^2+^ concentrations, initial pH, *Gracilaria* seaweed biomass (biosorbent), methylene blue concentrations and contact time. As shown in Table 1, each variable was evaluated at three coded levels (− 1, 0 and + 1). All variables, actual and coded levels and the full experimental design matrix with regard to variables levels are provided in Table 1. The responses values (Y) for methylene blue removal (%) and Ni^2+^ removal (%) in each trial were the average of the triplicate. All experiments were performed at 30 °C to save energy.

The correlations between the selected independent variables and the responses (methylene blue and nickel biosorption percentages) were determined using the equation of second-degree polynomial as follows:

3 $$Y = \beta_{0} + \sum\limits_{i} {\beta_{i} X_{i} } + \sum\limits_{ii} {\beta_{ii} X_{i}^{2} } + \sum\limits_{ij} {\beta_{ij} X_{i} X_{j} }$$

In which Y is the predicted methylene blue or nickel biosorption, X_i_ is the coded levels of the process parameters, β_i_ (linear coefficient), β_0_ (regression coefficients), β_ij_ (interaction coefficients) and β_ii_ (quadratic coefficients).

### Statistical analysis

“Design Expert version 7 for Windows and STATISTICA softwares were used for the experimental designs, statistical analysis and to plot the three-dimensional surface plots”.

### Analytical methods

The content of each flask for FCCCD experiment was centrifuged at 6000 × *g* and analyzed using Atomic Absorption spectroscopy according to “standard methods for the examination of water and wastewater 23rd edition 2017”^89^. The capacity of *Gracilaria* seaweed biomass as biosorbent for Ni^2+^ removal was determined as follows:

4 $${\text{Removal efficiency }}(\% ) = \frac{{{\text{C}}_{{\text{i}}} - {\text{C}}_{{\text{f}}} }}{{{\text{C}}_{{\text{i}}} }} \times 100$$

where, C_i_, C_f_ are the initial and final concentrations of nickel ions (mg/L); respectively.

The residual methylene blue concentrations were determined spectrophotometrically with a UV/Vis spectrophotometer at the wavelength of the highest absorbance (λ_max_) that was 670. The methylene blue removal % was determined as follows:

5 $${\text{Removal efficiency }}(\% ) = \frac{{{\text{C}}_{{\text{i}}} - {\text{C}}_{{\text{f}}} }}{{{\text{C}}_{{\text{i}}} }} \times 100$$

where, C_i_ and C_f_ are the initial and residual concentrations of methylene blue (mg/L); respectively.

### Fourier-transform infrared (FTIR) spectroscopy

FTIR analyses were performed in order to elucidate the distinctive surface functional groups of *Gracilaria* seaweed biomass that may be responsible for binding of methylene blue and nickel ions. The samples of *Gracilaria* seaweed dry biomass were analyzed before and after methylene blue and nickel ions biosorption with the FTIR spectroscopy (Thermo Fisher Nicolete IS10, USA spectrophotometer). The dry biomass samples of *Gracilaria* seaweed mixed with pellets of potassium bromide. The FTIR spectra were analyzed within the range of 400–4000 cm^−1^.

### Cell wall surface analysis by scanning electron microscopy (SEM)

The algal cell surface of dehydrated *Gracilaria* seaweed biomass samples were coated with gold and examined by SEM before and after the biosorption process.

## Conclusions

*Gracilaria* seaweed biomass shows high biosorption capacity for nickel and methylene blue dye. The optimal conditions for the maximum simultaneous removal percentages of the nickel ions and methylene blue were the algal biomass concentration of 6.94, initial pH level of 7.76, initial nickel ions concentration of 154.67 mg/L, initial concentration of methylene blue of 24.29 mg/L and contact time of 177.88 min. The experimental removal percentages of nickel ions and methylene blue were 98.9% and 97.31; respectively. It is recommended to use *Gracilaria* seaweed biomass for simultaneous removal of nickel and methylene blue dye from wastewaters generated from dyes industries.
